# Understanding the Role of Voluntary Counseling and Testing (VCT) in HIV Prevention in Nantong, China

**DOI:** 10.1155/2020/5740654

**Published:** 2020-10-05

**Authors:** Zhengcheng Xu, Ping Ma, Minjie Chu, Yujia Chen, Junyan Miao, Hongli Xia, Xun Zhuang

**Affiliations:** ^1^School of Public Health, Nantong University, No. 9 Seyuan Road, Nantong, Jiangsu Province 226000, China; ^2^Nantong Centre for Disease Control and Prevention, No. 189 Gongnong Road, Nantong, Jiangsu Province 226000, China

## Abstract

Voluntary counseling and testing (VCT) service plays an essential part in the prevention of human immunodeficiency virus (HIV) infection. The purpose of this study was to investigate the characteristics of participants and analyze the major factors of HIV infection in VCT in Nantong, China. This study was conducted between January 2010 and December 2015, based on the responses to questionnaires and blood test results retrieved from the Chinese National HIV/AIDS Comprehensive Control Information System (CNHCCIS). Multivariate logistic regression analyses were used to identify factors related to HIV infection. Differences between first-time testers and repeat testers were assessed using the chi-squared or Fisher test. Over six years, a total of 11,560 VCT participants were included, and 420 cases were confirmed to be HIV-positive. Overall, the annual number of participants was relatively stable with a mean of 1927, while there was a rapid increase in the HIV detection rate (from 1.03% in 2010 to 7.52% in 2015). In multivariate analysis, referral counseling and having a HIV-positive spouse/fixed sex partners were found to be significantly associated with HIV infection among all participants, while being unmarried or divorced, having commercial heterosexual behaviors, and male-male sexual behaviors are additional HIV-related factors for males. Compared to first-time testers, repeat testers were more willing to engage in high-risk sexual behaviors and had higher HIV detection rates (*P* < 0.001). In conclusion, the HIV epidemic in Nantong is still not controlled. Therefore, in the future, it is critical to expand VCT services to increase the detection rate of HIV, which can prevent the transmission of HIV effectively.

## 1. Introduction

From 2014, more than 10,000 people have been infected with human immunodeficiency virus (HIV) every year in China [1]. By the end of August 2018, 841,478 people in China were estimated to be living with HIV, and 226,557 were reported to have died due to HIV [2, 3]. HIV is not only a health concern, but it also has social, economic, and political implications. Data from China showed that more than 56% of people living with HIV (PLWH) were unaware of their infection [4]. Therefore, it is more important to identify and confirm the infection status at the earliest.

In 1994, the World Health Organization (WHO) and the Joint United Nations Program on HIV/AIDS (UNAIDS) proposed a strategy called HIV voluntary counseling and testing (VCT) to prevent and control HIV/AIDS. VCT provided an opportunity for both a primary prevention (i.e., preventing HIV-negative people from contracting the infection) and a secondary prevention (i.e., avoiding the progression of the disease in infected people by providing early health care and psychosocial support) as it encompasses counseling before and after HIV testing [5]. VCT had been widely accepted as the cornerstone of HIV prevention programs in many countries because of its multiple benefits [6].

The world had committed to end the HIV epidemic by 2030 as a part of the Sustainable Development Goals. Therefore, China had the responsibility to achieve the goal as well. In 2004, a free VCT program was introduced in China, and the number of VCT clinics was rising every year [7]. By June 2016, 588,970 people had received VCT, and 580,974 people underwent HIV-antibody testing, and 12,340 HIV-antibody positive individuals were detected in VCT, which accounted for 3.4% among all HIV-infected people in China [8]. An epidemiological modelling study conducted in 2012 found that a fourfold increase in VCT would avert 42,000 cases of HIV and 11,000 HIV-related deaths over the next five years in China [9]. Therefore, scaling up the coverage of HIV testing in China was recognized as an important strategy for identifying unknown positives and preventing the onward transmission of HIV.

In addition, increasing repeat HIV testing, as a goal of VCT-clinic-based interventions, was also widely encouraged to achieve timely HIV detection among people at high risk for HIV infection [10]. Moreover, tailored health education from VCT clinics can help VCT participants reduce risk behaviors and protect themselves. However, the effectiveness of repeated VCTs on behavioral risk reduction was mixed [11]. Some studies showed that more risk behaviors were found among repeat testers compared with first-time testers [12, 13]. The reason of these unexpected results and difference in characteristics between first-time testers and repeat testers are needed to be investigated to enhance the effectiveness of VCT.

In Jiangsu Province, eastern China, 96,878 free HIV counseling and 95,348 free testings had been implemented by the end of 2014 [14]. Nantong is located in the central part of Jiangsu, which reported the first HIV-infected person in 1998 [15]. VCT has been carried out in Nantong since 2004 [16]. Our study was aimed at describing the characteristics of VCT participants from 2010 to 2015, analyze the HIV-related factors, and compare the differences between first-time and repeat testers. We tried to uncover the main transmission routes of HIV and the characteristics of HIV-positive people in Nantong. This information is essential to control local HIV prevalence. Furthermore, we hope the present study will provide insights into enhancing the coverage and effectiveness of VCT services.

## 2. Materials and Methods

### 2.1. Field of Study and Data Collection

Nantong is located in the south-eastern part of China, with 7.80 million inhabitants. The study was performed between January 2010 and December 2015 in the Centers for Disease Control and Prevention (CDC) of eight districts (Qidong, Rugao, Haimen, Haian, Rudong, Chongchuan, Gangzha, and Tongzhou) in Nantong. VCT clinics were open to every participant and provided free HIV/syphilis testing at all times.

VCT participants were interviewed by physicians face to face with the help of questionnaires. All physicians had a professional background of medical or public health. They were capable of providing HIV-related health education and elaborating on the meanings of the HIV test results.

The questionnaire included four parts, namely, social demographics (age, gender, education, etc.), counseling sources, counseling reasons, and HIV testing history. The counseling sources consisted of active counseling, outreach service for high-risk groups, and referral counseling. Active counseling referred to people who went to voluntarily to VCT clinics for counseling and testing. Outreach service targeted participants from places of entertainment such as bathhouses, massage centers, salons, bars, and hotels. Participants from referral counseling refer to those people who were suggested by local medical institutions to undergo an HIV test for confirmation at CDC because (1) they had positive results at their preliminary HIV screening test or (2) their clinical symptoms showed high risk of HIV infection. Further, the counseling reasons included (1) injected drug use (IDU), (2) commercial heterosexual behavior, (3) noncommercial/nonfixed heterosexual behavior, (4) having an HIV-positive spouse/fixed sexual partners; (5) male-male sexual behavior, (6) blood transmission, (7) no high-risk behavior, and (8) others. Each participant could choose one option based on their latest risky behaviors. Moreover, physicians also assessed HIV testing history to determine whether subjects had undergone HIV testing in the past. Repeat testers referred to those who had undergone testing for HIV before. Participants who tested positive for either HIV or other STIs obtained related referral services from VCT clinics.

All information in the questionnaire was entered into the VCT information module in the Chinese National HIV/AIDS Comprehensive Control Information System (CNHCCIS). Data from eight local CDCs during January 2010 to December 2015 were retrieved from CNHCCIS in our study. Inclusion criteria for the study were as follows: all participants who were willing to take part in the VCT survey. Willing participants who were intoxicated or mentally ill to the extent that they could not consent to study participation were excluded.

This study was approved by the Ethics Committee of Nantong CDC. As this was a retrospective analysis of deidentified records which did not include personally identifying information, informed consent was not required.

### 2.2. Laboratory Testing

Five ml venous blood was collected from each VCT participant, and all samples were transferred to the CDC laboratory for testing for HIV and syphilis antibodies. Enzyme-linked immunosorbent assay was used for HIV screening, and positive samples were confirmed by Western blot analysis. Syphilis serology was determined through rapid plasma reagin (RPR), and RPR-positive samples were subjected to confirmatory testing using the Treponema pallidum particle agglutination assay.

### 2.3. Data Analysis

All statistical analyses were performed using SPSS 24.0 (IBM Corporation, Chicago, IL). Frequency and percentage were used to describe the distribution of each variable. Chi-squared or Fisher test was applied to analyze the disparity between two categorical variable groups. Univariate and multivariate logistic regression analyses were used to analyze the HIV-related factors. All variables with *P* < 0.10 in the univariate logistic regression model were included in the following multivariate regression analysis. Univariate and multivariate logistic regression was used to estimate the adjusted odds ratios (aOR) and 95% confidence intervals (CI). *P* < 0.05 was considered statistically significant.

## 3. Results

### 3.1. General Characteristics of the Study Participants

From January 2010 to December 2015, none of the VCT clients was excluded and data of 11,560 individuals were included for this study. Among the participants, the mean age was 33.17 ± 10.01 years. A majority of them were 20–39 years old (71.21%), male (53.75%), and currently married (67.02%). Participants who had received a junior high school education or lower accounted for 70.07%. Of the total, 8723 (75.46%) individuals were from active counseling, 1993 (17.24%) individuals had never undergone testing for HIV before, and 9062 participants underwent syphilis test this time of which the positivity rate was 1.05% (95/9062). The prevalence of HIV was 3.63% (420/11560). Most participants went for VCT after risky sexual behavior, and the main counseling reason (59.87%) was participation in commercial heterosexual behavior in the past (see Table 1).

**Figure 1:**
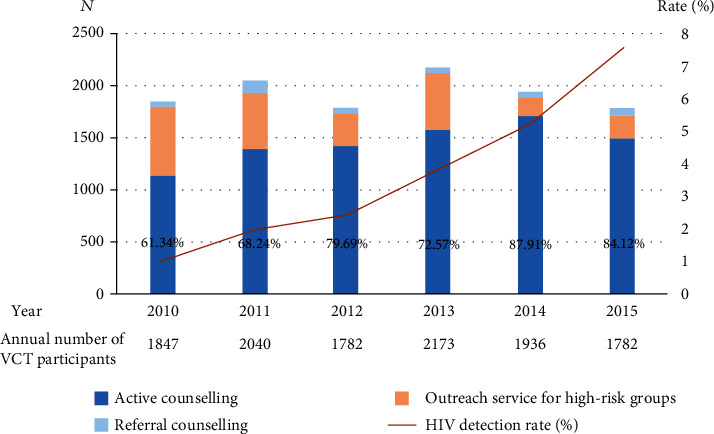
Trending of VCT participants and HIV detection from 2010 to 2015. The annual number of participants was relatively stable, while there was a rising tendency in the HIV detection rate and the proportion of active counseling.

The annual number of VCT participants fluctuated slightly through 2010–2015. However, the number of HIV-positive individuals increased every year (19, 2010; 40, 2011; 43, 2012; 83, 2013; 101, 2014; and 134, 2015). Additionally, the detection rate of HIV-positive participants showed an obvious rising tendency with years (*χ*^2^ = 142.54, *P* < 0.05). Moreover, there was an upward trend in the proportion of active counseling (*χ*^2^ = 288.44, *P* < 0.05) and repeat testers each year (*χ*^2^ = 280.58, *P* < 0.05) (see Figure 1).

### 3.2. Differences in the Characteristics of HIV-Positive and HIV-Negative Participants

There were 420 (3.63%) people who were confirmed to be HIV-positive among the 11,560 participants, and most of them were male (91.34%), 20–39 years old (62.14%), and currently married (45.48%); and 181 (43.10%) people received junior high school or lower education, and 82.38% were from active counseling. Besides, 67.14% of HIV-positive participants visited the VCT as they engaged in male-male sexual behavior (see Table 1). As compared to HIV-negative participants, the HIV-infected group had a higher proportion of males (*χ*^2^ = 248.95, *P* < 0.001) and individuals with unmarried/divorced/widowed status (*χ*^2^ = 239.14, *P* < 0.001). The percentage of HIV-positive individuals who received senior high school or higher education (56.91%) was much higher than that of HIV-negative individuals (28.91%) (*χ*^2^ = 152.31, *P* < 0.001). Male-male sexual behavior (67.14%) accounted for the largest proportion among the counseling reasons of the HIV-positive group. However, 61.43% of the HIV-negative groups underwent counseling because of engaging in commercial heterosexual behaviors. It was apparent that the proportion of each counseling reason was different in two groups (*χ*^2^ = 1861.04, *P* < 0.05). Most people (82.38% in the HIV-positive group, 75.20% in the HIV-negative group) participated in VCT actively for counseling, but the percentage of referral counseling was much higher among the HIV-positive group (17.14% vs 2.74%). In addition, HIV-positive participants were more willing to test for syphilis in comparison with HIV-negative participants (92.38% vs. 77.87%; *χ*^2^ = 86.67, *P* < 0.001).

The result of multivariable logistic regression analyses of HIV-positive status among females and males is presented in Table 2. In females, those who had HIV-positive spouses/fixed sex partners (aOR = 6.85, *P* < 0.001) were more likely to be infected with HIV. However, the aOR of HIV infection with commercial heterosexual behavior as the counseling reason was 0.04 (*P* < 0.01). The reason may be that among 36 HIV-positive female participants in our study, only one came for having commercial heterosexual behaviors. In males, sociodemographic factors independently associated with HIV infection were age and marital status. The odds of HIV infection among male participants who were unmarried (aOR = 2.40, *P* < 0.001) and divorced (aOR = 3.54, *P* < 0.001) were higher than those among married individuals. Furthermore, males who had commercial heterosexual behavior (aOR = 1.80, *P* < 0.05) and male-male sexual behavior (aOR = 25.50, *P* < 0.001) were more likely to contract HIV. Compared with participants from active counseling, participants from referral counseling were more likely to be HIV-positive.

### 3.3. Differences in the Characteristics of Repeat Testers and First-Time Testers

Among 11560 participants, 9567 underwent testing for HIV the first time, and the other 1993 had undergone testing before. The proportion of male participants was higher in repeat testers compared to first-time testers (61.87% vs. 52.05%, *P* < 0.001). Among the repeat testers, 49.42% had attained secondary school education, higher than their counterparts (25.81%) (*χ*^2^ = 490.64, *P* < 0.001). Additionally, there was an obvious difference in counseling reasons among the two groups (*χ*2 = 1047.35, *P* < 0.001). Most participants of both the groups sought VCT as they had engaged in commercial heterosexual behaviors (40.54% vs. 63.90). However, compared with first-time testers, more repeat testers came for engaging in male-male sexual behaviors (23.53% vs. 5.82%) and HIV-positive spouse/fixed sex partners (13.30% vs. 3.70%). Furthermore, the HIV detection rate of repeat testers (8.38%) was relatively higher than the other group (2.64%), and nearly 40% HIV-positive participants had tested for HIV before. Repeat testers also were more willing to undergo syphilis testing, and the positivity rate was also higher than first-time testers (1.46% vs. 0.69%, *χ*2 = 103.59, *P* < 0.001) (see Table 3).

## 4. Discussion

Our study showed that the average HIV-positivity rate among VCT participants in Nantong was 3.63%, which was higher than the nationwide infection rate (2.12%) [3]. The annual number of VCT participants was relatively stable in Nantong city from 2010 to 2015; however, the HIV infection rate rose steadily through the years, indicating that there was a potentially rising prevalence of HIV in Nantong in recent years. Moreover, it was noteworthy that the trend between the annual number of VCT participants and HIV infection rate was found to be inconsistent, which was also observed in other cities in China, such as Shenzhen [17] and Linyi [18]. This could remind us that the acceptance of VCT was unexpectedly low in some areas of China, despite the essential role it played in facilitating the early detection of HIV infection [19].

Furthermore, in Nantong, the youth (20–40 years old) accounted for nearly 70% of all VCT participants, similar to Shenzhen [17]. People tended to be sexually active in the period of youth [20]. Additionally, young people were more likely to practice risky sexual behaviors due to inadequate education and negative attitudes towards HIV [21]. Sex education is an important part of HIV prevention; hence, more interventions should be implemented to disseminate HIV-related knowledge among the youth. In addition, VCT participants underwent counseling mainly because they had commercial heterosexual behaviors, as reported by some papers [19, 22]. A study showed that the frequency of condom use among people engaging in commercial sexual behaviors was low, which could greatly increase the risk of HIV infection [23].

In the HIV-positive group, people older than 40 years accounted for 34.76%, which is notable, and 41.78% of them were older than 50 years. In China, there was a significant increase in the number of new HIV infections in individuals over 50 years of age [2]. A similar situation has been observed worldwide. More than 50% of PLWH in Australia were estimated to be over 50 years of age [24]. Furthermore, in the United Kingdom, the number of old people newly diagnosed with HIV was increasing despite the decrease in overall rates of new HIV diagnoses [25]. The reason may be that old people had lower HIV-related knowledge and risk perception [26]. In fact, evidence showed that many older people remained sexually active despite the public preconception about a lack of sexual behaviors in this group [27]. Moreover, we found that 54.52% of HIV-positive testers were unmarried and divorced or widowed, and this may be a reason why they were more likely to engage in commercial sexual behaviors [28]. The loss of a spouse could be viewed as the factor influencing the transition to high-risk sexual behaviors as people leave settled relationships and reach a phase during which they may look for new sexual partners.

Furthermore, with respect to MSM (men who have sex with men), it was the dominant group (67.14%) among HIV-positive persons in our study. Male-male sexual behaviors had apparently become one of the main routes of HIV transmission in mainland China, and the HIV prevalence among Chinese MSM was approximately 8% in 2015 [29]. To bring about a decrease in the striking prevalence of HIV infection in MSM, it is essential to expand the coverage of HIV testing as well as counseling on risk reduction. Novel and feasible HIV screening methods, such as HIV self-testing, have been widely promoted in order to increase the rate of diagnosis among MSM [30]. Studies show that HIV self-testing is highly acceptable among MSM in some countries [31, 32]. However, its uptake rate is relatively low in China, which is only 15.6%-27%. The reason maybe be the limited availability of counseling and self-test information in China [33, 34]. Research has found that MSM prefer to undergo VCT for HIV testing because of anonymous and specialized HIV-related counseling services [35]. Obviously, VCT still plays an indispensable role in HIV testing and prevention among MSM. Moreover, it is recommended that MSM should take HIV testing at least once per year for their high-risk behaviors [36]. Therefore, encouraging MSM to test for HIV more actively and promoting other approaches of HIV testing will be necessary to increase the detection rate.

On comparing the characteristics of repeat testers and first-time testers, we found that repeat testers had higher education levels. However, the people of this group were more likely to engage in some high-risk sexual behaviors, and they were at a higher risk of HIV infection. This phenomenon could be found in other studies as well [12, 37]. The reason may be that negative results of repeated VCT would provide VCT participants a false sense of security to some extent. In other words, they are likely to view negative VCT results as a positive signal that indicating that their ongoing risk behaviors are not dangerous enough to result in an HIV infection [38], and this notion could foster greater risk in the future [12]. Hence, physicians should pay special attention to those who have already undergone VCT and provide specific and personalized health counseling to help repeat testers get rid of this inappropriate notion, which is crucial to enhance the quality of VCT.

Another noticeable aspect was that the percentage of referral counseling was much higher among the HIV-positive group. The reason may be that those participants whose blood samples tested positive in the hospital would be asked to take a VCT in a local CDC for further verification. Undoubtedly, this move can enhance the HIV detection rate in VCT. Therefore, strengthening the communication between VCT clinics and other local community and health services is also an indispensable part of HIV prevention work.

Furthermore, HIV testing rates among MSM and other high-risk groups such as female sexual workers in China remained low as well, mainly due to unawareness of how to seek VCT services and due to HIV stigma [39]. HIV stigma had been observed in China, and the fear of stigma and public discrimination was a barrier to HIV prevention and care efforts [19]. In order to promote HIV testing, it is crucial to improve VCT acceptance and disseminate HIV-related knowledge. For this, several ways could be used. Internet-facilitated methods, such as short messages and emails [40], can be used to conduct health education and promote timely testing among high-risk groups. Additionally, peer education [41] and community-based VCT [42] are also effective propagation methods. With an expansion in the scope of VCT implementation, we will enhance the detection rate of HIV and enable more HIV-infected people to receive timely antiretroviral therapy.

There were some limitations in our study. As our study included all testing data in Nantong from 2010 to 2015, the data can only reflect the HIV prevalence situation in Nantong to some extent. Since the objective of our study was to cover only the VCT participants, the results may not represent the general population. Furthermore, the data was collected by face-to-face interviews; therefore, there could have been information bias due to recall bias, the sensitive nature of the questions, and social stigma factors. Additionally, counseling reasons were used to discover main transmission routes of HIV among all VCT participants, while this measurement may not represent sexual behaviors accurately.

## 5. Conclusions

Our study described the characteristics of 11,560 VCT participants during 2010–2015 and indicated that the HIV detection rate of the VCT service was increasing with years. Thus, it is important to scale up VCT services to screen and identify more unknown HIV-positive individuals Moreover, the present study can help us understand the role of VCT in HIV prevention and provide insights into enhancing the coverage and effectiveness of VCT services.

## Figures and Tables

**Table 1:** Comparing characteristics of HIV-positive VCT participants and HIV-negative VCT participants from 2010 to 2015 in Nantong, China.

Characteristics	Total (*n* = 11,560) *n* (%)	HIV-positive (*n* = 420) *n* (%)	HIV-negative (*n* = 11,140) *n* (%)	*χ* ^2^	*P*
Gender				248.95	<0.001
Female	5347 (46.25)	36 (8.57)	5311 (47.68)		
Male	6213 (53.75)	384 (91.43)	5829 (52.32)		
Age (years)				50.81	<0.001
<20	427 (3.69)	13 (3.10)	414 (3.72)		
20-	4426 (38.29)	156 (37.14)	4270 (38.33)		
30-	3806 (32.92)	105 (25.00)	3701 (33.22)		
40-	2133 (18.45)	85 (20.24)	2048 (18.38)		
50-	597 (5.16)	46 (10.95)	551 (4.95)		
≥60	171 (1.48)	15 (3.57)	156 (1.40)		
Marital status				239.14	<0.001
Married	7748 (67.02)	191 (45.48)	7557 (67.84)		
Unmarried	3552 (30.73)	178 (42.38)	3374 (30.29)		
Divorced or widowed	260 (2.25)	51 (12.14)	209 (1.88)		
Education level				152.31	<0.001
Junior high school or lower	8100 (70.07)	181 (43.10)	7919 (71.09)		
Senior high school or secondary school	1763 (15.25)	116 (27.62)	1647 (14.78)		
College or higher	1697 (14.68)	123 (29.29)	1547 (14.13)		
Counseling source				348.94	<0.001
Active counseling	8723 (75.46)	346 (82.38)	8377 (75.20)		
Outreach services for high-risk groups	2460 (21.28)	2 (0.48)	2458 (22.06)		
Referral counseling	377 (3.26)	72 (17.14)	305 (2.74)		
Counseling reasons				1861.04	<0.001
Noncommercial/nonfixed heterosexual behaviors	2290 (19.81)	27 (6.43)	2263 (20.31)		
Commercial heterosexual behaviors	6921 (59.87)	78 (18.57)	6843 (61.43)		
HIV-positive spouse/fixed sex partners	619 (5.35)	31 (5.01)	588 (5.28)		
Male-male sexual behaviors	1026 (8.88)	282 (67.14)	744 (6.68)		
IDU (injected drug use)	25 (0.22)	2 (0.48)	23 (0.21)		
Blood transmission	116 (1.00)	0 (0.00)	116 (1.04)		
No high-risk behavior	432 (3.74)	0 (0.00)	432 (3.88)		
Others^a^	131 (1.13)	0 (0.00)	131 (1.18)		
Ever tested for HIV				154.93	<0.001
No	9567 (82.76)	253 (60.24)	9314 (83.61)		
Yes	1993 (17.24)	167 (39.76)	1826 (16.39)		
Having a syphilis antibody test this time				86.67	<0.001
No	2498 (21.61)	32 (7.62)	2466 (22.14)		
Yes, syphilis antibody (+)	95 (0.82)	15 (3.57)	80 (0.72)		
Yes, syphilis antibody (-)	8967 (77.57)	373 (88.81)	8594 (77.15)		

^a^Others include rejecting telling the enquiry reasons, history of taking care of the HIV^+^ person, and father HIV-positive history.

**Table 2:** Results of logistic regression of factors associated with HIV infection among females and males.

Characteristics	Female (*N* = 5347)		Male (*N* = 6213)	
	cOR	aOR	cOR	aOR
Age (years)				
≥60	1	1	1	1
<20	-	-	0.41 (0.18-0.92)^∗^	0.09 (0.03-0.24)^∗∗∗^
20-	0.12 (0.25-0.57)^∗∗^	1.95 (0.30-12.73)	0.53 (0.29-0.97)^∗^	0.20 (0.09-0.44)^∗∗∗^
30-	0.10 (0.02-0.49)^∗∗^	1.28 (0.20-8.12)	0.40 (0.22-0.73)^∗∗^	0.37 (0.17-0.78)^∗∗^
40-	0.25 (0.05-1.21)	1.09 (0.18-6.49	0.48 (0.26-0.90)^∗^	0.51 (0.24-1.10)
50-	1.91 (0.42-8.80)	3.31 (0.59-18.54)	0.69 (0.35-1.36)	1.05 (0.46-2.40)
Marital status				
Married	1	1	1	1
Unmarried	0.27 (0.10-0.78)^∗^	0.94 (0.22-4.08)	2.78 (2.23-3.47)^∗∗∗^	2.40 (1.63-3.53)^∗∗∗^
Divorced or widowed	4.84 (1.67-12.06)^∗∗^	2.09 (0.57-7.44)	11.04 (7.57-16.08^)∗∗∗^	3.54 (2.22-5.66)^∗∗∗^
Education level				
Junior high school or lower	1	1	1	1
Senior high school or secondary school	2.37 (1.07-5.27)^∗^	1.23 (0.51-2.95)	2.25 (1.74-2.90)^∗∗∗^	1.24 (0.89-1.72)
College or higher	1.35 (0.32-5.70)	0.48 (0.09-2.54)	2.04 (1.60-2.61)^∗∗∗^	0.83 (0.59-1.18)
Counseling source				
Active counseling	1	1	1	1
Outreach services for high-risk groups	0.10 (0.02-0.41)^∗∗^	0.91 (0.20-4.17)	-	-
Referral counseling	6.94 (3.26-14.75)^∗∗∗^	27.55 (9.91-76.59)^∗∗∗^	7.78 (5.65-10.70)^∗∗∗^	5.44 (3.58-8.25)^∗∗∗^
Counseling reasons				
Noncommercial/nonfixed heterosexual behaviors	1	1	1	1
Commercial heterosexual behaviors	0.03 (0.003-0.22)^∗^	0.04 (0-0.32)^∗∗^	1.87 (1.13-3.11^)∗^	1.80 (1.80-3.00)^∗^
HIV-positive spouse/fixed sex partners	5.31 (2.38-11.81)^∗∗∗^	6.85 (2.28-20.56)^∗∗∗^	4.12 (1.50-11.27)^∗∗^	3.33 (1.18-9.37)^∗^
Male-male sexual behaviors	-	-	29.03 (18.09-46.58)^∗∗∗^	25.50 (15.40-42.21)^∗∗∗^
IDU (injected drug use)	25.25 (2.53-151.67)^∗^	27.33 (4.72-113.70)^∗∗^	4.03 (0.51-31.66)	1.50 (0.14-9.62)
Ever tested for HIV				
No	1	1	1	1
Yes	4.38 (2.25-8.53)^∗∗∗^	0.86 (0.40-1.86)	2.88 (2.32-3.57)^∗∗∗^	0.95 (0.72-1.24)
Having a syphilis antibody test this time				
No	1	1	1	1
Yes, syphilis antibody (+)	-	-	9.70 (4.87-19.31^)^^∗∗∗^	4.34 (1.89-9.99)^∗∗^
Yes, syphilis antibody (-)	4.72 (1.45-15.42)^∗^	2.47 (0.61-10.07)	2.11 (1.43-3.10)^∗∗∗^	1.61 (1.04-2.50)^∗^

cOR: univariate odds ratio; aOR: multivariate odds ratio obtained from “forward” stepwise logistic regression using variables *P* < 0.10 in univariate analysis as candidates (entry: *P* < 0.10). Associations between the factors and HIV positive were tested by logistic regression models, adjusted for all the factors in this table. ^∗^*P* < 0.05; ^∗∗^*P* < 0.01; ^∗∗∗^*P* < 0.001. “-”: the number of HIV-positive cases in this group is 0.

**Table 3:** Comparing characteristics of repeated testers and first-time testers.

Characteristics	Repeat testers (*n* = 1993)	First-time testers (*n* = 9567)	*χ* ^2^	*P*
Year			280.58	<0.001
2010	259 (13.00)	1588 (16.60)		
2011	214 (10.74)	1826 (19.09)		
2012	265 (13.30)	1517 (15.86)		
2013	254 (12.74)	1919 (20.06)		
2014	407 (20.42)	1529 (15.98)		
2015	594 (29.80)	1188 (12.42)		
Gender			63.89	<0.001
Female	760 (38.13)	4587 (47.95)		
Male	1233 (61.87)	4980 (52.05)		
Age			41.11	<0.001
<20	67 (3.36)	360 (3.76)		
20-	871 (43.70)	3550 (37.16)		
30-	581 (29.15)	3225 (33.71)		
40-	336 (16.86)	1797 (18.78)		
50-	95 (4.77)	502 (5.25)		
≥60	43 (2.16)	128 (1.34)		
Marital status			106.34	<0.001
Married	656 (32.92)	2875 (30.05)		
Unmarried	1241 (62.27)	6493 (67.87)		
Divorced or widowed	96 (4.82)	199 (2.08)		
Education level			490.64	<0.001
Junior high school or lower	1008 (50.58)	7092 (74.19)		
Senior high school or secondary school	420 (21.07)	1343 (14.04)		
College or higher	565 (28.35)	1132 (11.83)		
Counseling source			211.03	<0.001
Active counseling	1570 (78.78)	7153 (74.77)		
Outreach services for high-risk groups	271 (13.60)	2189 (22.88)		
Referral counseling	152 (7.63)	225 (2.35)		
Counseling reasons			1047.35	<0.001
IDU (injected drug use)	9 (0.45)	16 (0.17)		
HIV-positive spouse/fixed sex partners	265 (13.30)	354 (3.70)		
Commercial heterosexual behaviors	808 (40.54)	6113 (63.90)		
Noncommercial/nonfixed heterosexual behaviors	314 (15.76)	1976 (20.65)		
Male-male sexual behaviors	469 (23.53)	557 (5.82)		
No high-risk behavior	88 (4.42)	344 (3.60)		
Blood transmission	4 (0.20)	11 (0.11)		
Others	36 (1.81)	196 (2.05)		
HIV status			154.93	<0.001
HIV-positive	167 (8.38)	253 (2.64)		
HIV-negative	1826 (91.62)	9314 (97.36)		
Having a syphilis antibody test			103.59	<0.001
Yes, syphilis antibody (+)	29 (1.46)	66 (0.69)		
Yes, syphilis antibody (-)	1696 (85.10)	7271 (76.00)		
No	268 (13.45)	2230 (23.31)		

## Data Availability

The data used to support the findings of this study have not been made available because data are saved in the Chinese National HIV/AIDS Comprehensive Control Information System.
